# Supplementary material for: Genomic analyses of rice bean landraces reveal adaptation and yield related loci to accelerate breeding
Source: Nat Commun. 2022 Sep 29;13:5707. doi: 10.1038/s41467-022-33515-2 (PMC9523027; doi:10.1038/s41467-022-33515-2)
Supplement: Supplementary file 1 — Supplementary Information [file 41467_2022_33515_MOESM1_ESM.pdf]

Genomic analyses of rice bean landraces reveal adaptation and yield related loci to accelerate breeding

Guan *et al.*

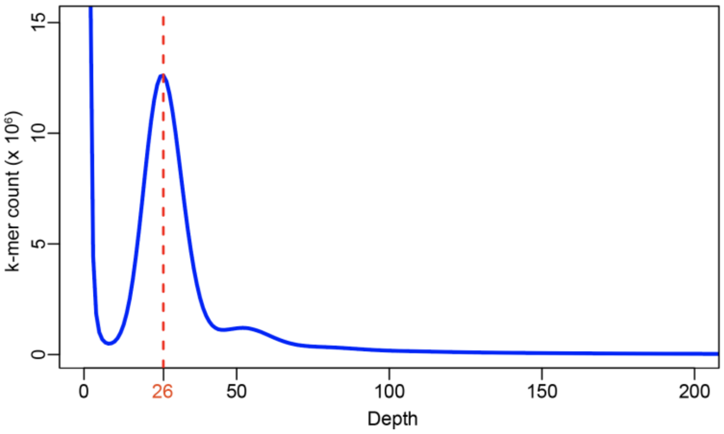

Supplementary Fig. 1. 17-mer depth distribution of the Illumina short-reads. The genome size of FF25 was 525.60 Mb, which was estimated based on the distribution of k-mer frequency and optimized using GCE v1.0.2.

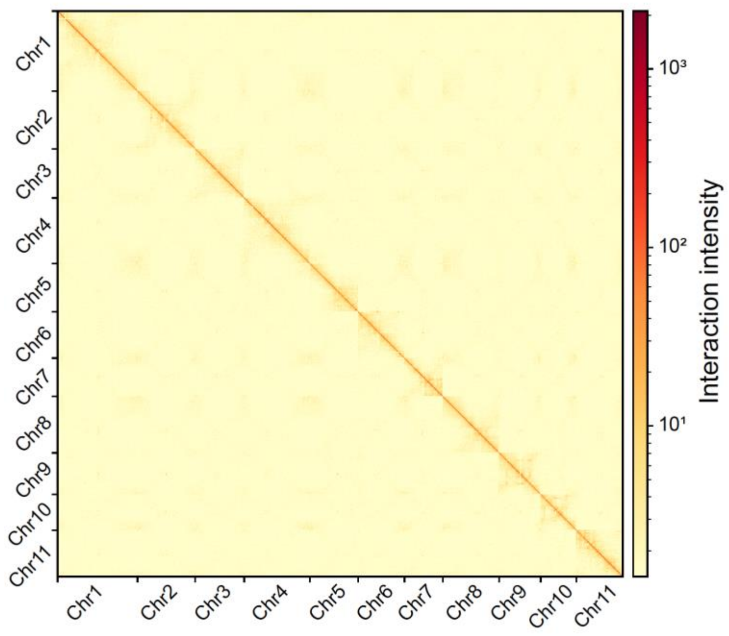

Supplementary Fig. 2. Genome-wide ICE (iterative correction and eigenvector decomposition)-corrected Hi-C interaction heatmaps at 40 Kb windows for the genome assembly. The diagonal lines represent the frequency of contact between two 40 Kb windows on a chromosome.

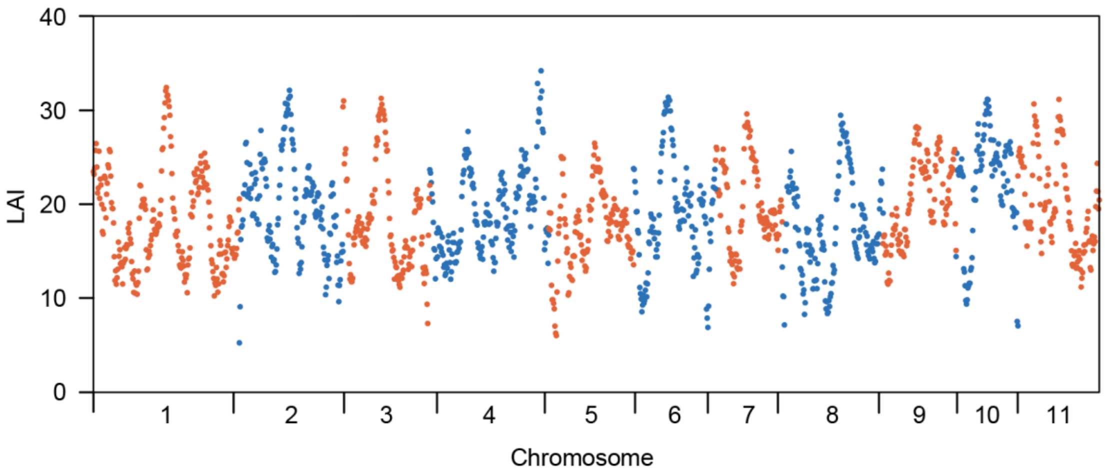

Supplementary Fig. 3. Genome-wide distribution of LAI (LTR Assembly Index) scores in 3Mb windows.

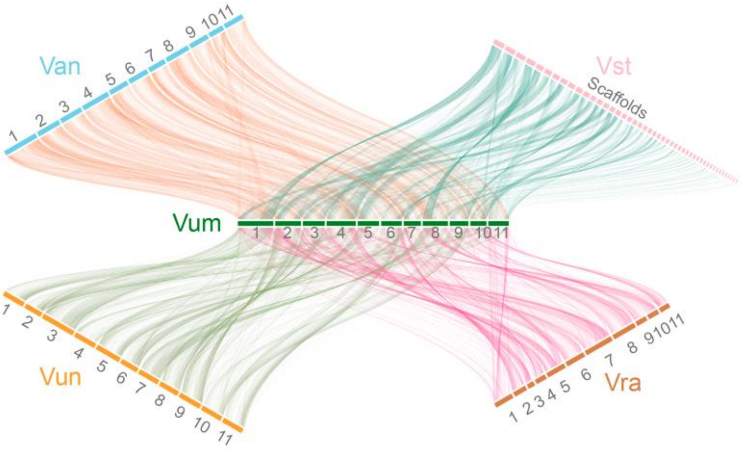

Supplementary Fig. 4. Syntenic genes between *Vigna umbellata* (Vum) and its closely related *Vigna* species (*V. unguiculate* (Vun), *V. angularis* (Van), *V. radiata* (Vra), and *V. stipulacea* (Vst)). Note that the Vst genome only shows the top 47 longest scaffolds (> 1 Mb).

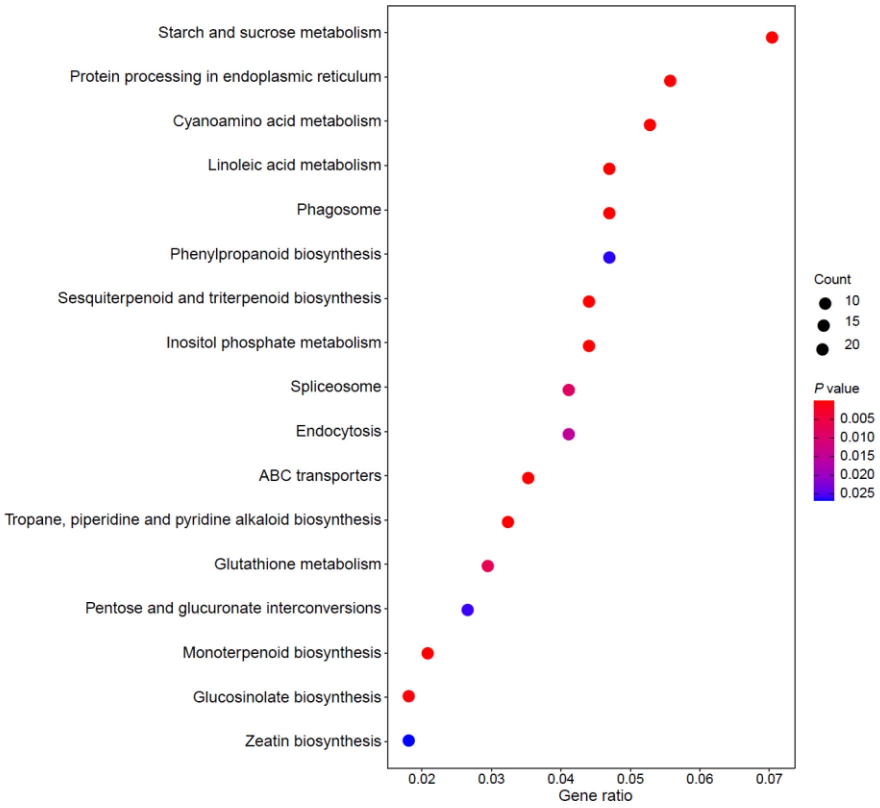

Supplementary Fig. 5. The KEGG enrichment analyses of 1,396 genes in 230 significantly expanded families of rice bean. Significance was tested with the two-sided Fisher's exact test. The significant term was the one with P value < 0.05.

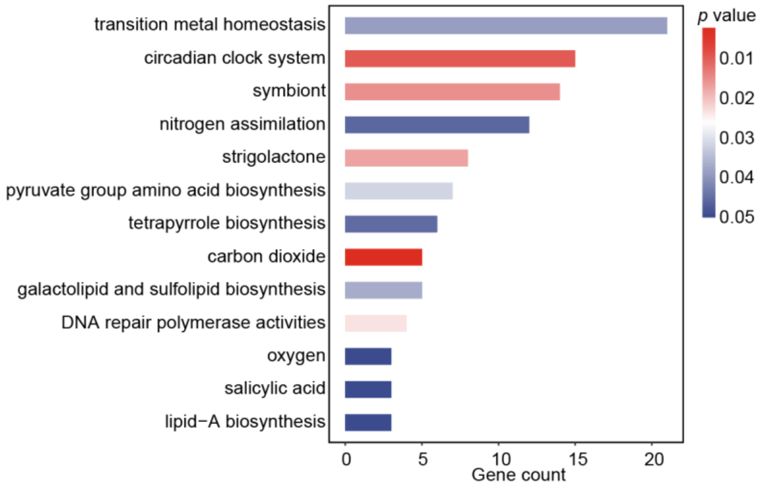

Supplementary Fig. 6. MapMan enrichment analysis of all the selected genes for the three comparisons (SSA vs. SC, SSA vs. NC, and SC vs. NC). Significance was tested with the two-sided Fisher's exact test. The significant term was the one with *P* value < 0.05.

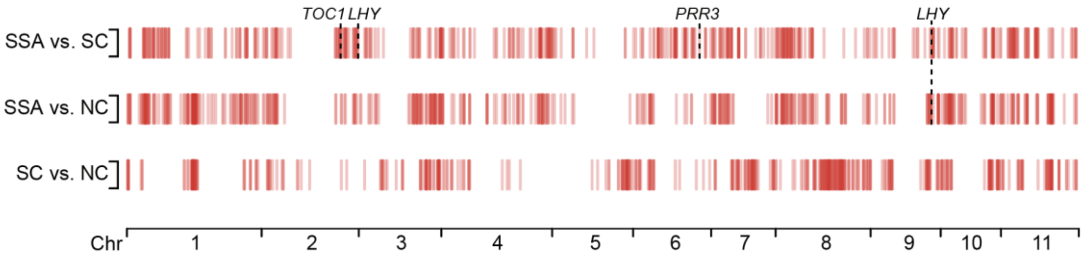

Supplementary Fig. 7. Genomic divergence signals for the three comparisons (SSA vs. SC, SSA vs. NC, and SC vs. NC) and homologous genes related to "circadian clock system" within these signals. The red lines represent the diverged genomic signals and the black dashed lines indicate the locations of the homologous genes related to "circadian clock system".

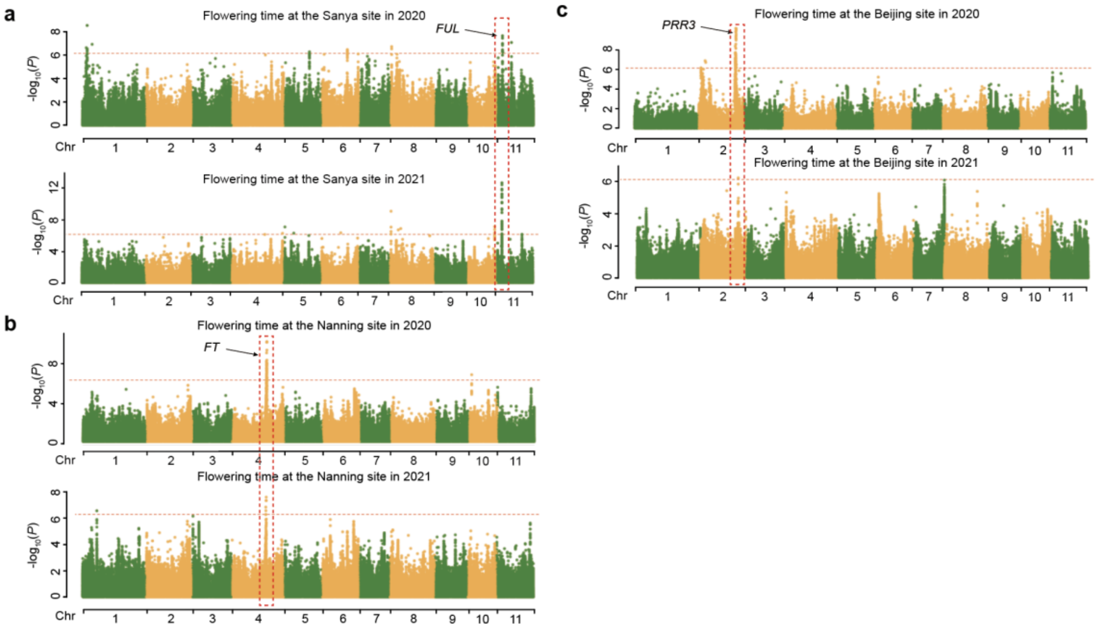

Supplementary Fig. 8. Manhattan plots of GWAS for flowering-time data measured at the Sanya (a), Nanning (b), and Beijing (c) sites across two years (2020 and 2021). Red horizontal dashed line indicated the Bonferroni-corrected significance thresholds of GWAS (α = 1).

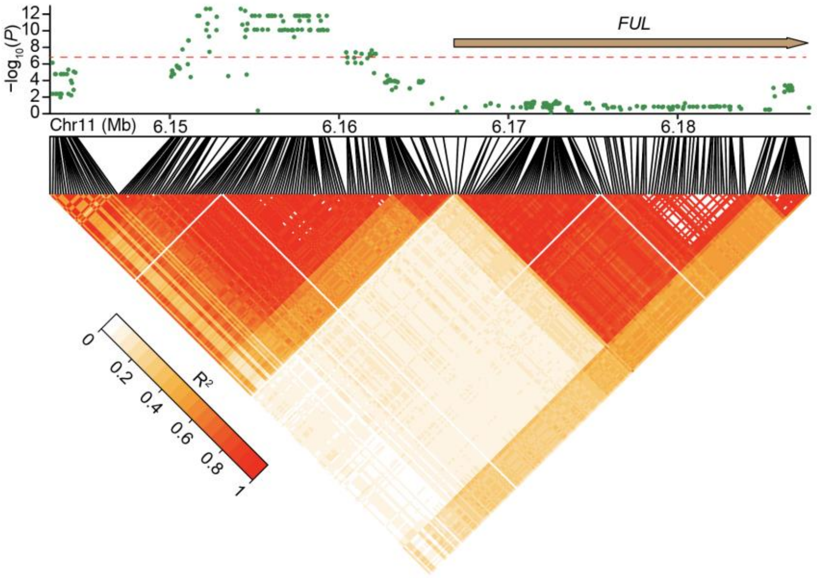

Supplementary Fig. 9. Local Manhattan plot and pairwise linkage disequilibrium (LD) heat map among SNPs located in the associated region with the flowering time measured in Sanya in 2021. The candidate protein-coding gene *FUL* is shown as colored arrows. Red horizontal dashed line indicated the Bonferroni-corrected significance thresholds of GWAS (α = 1).

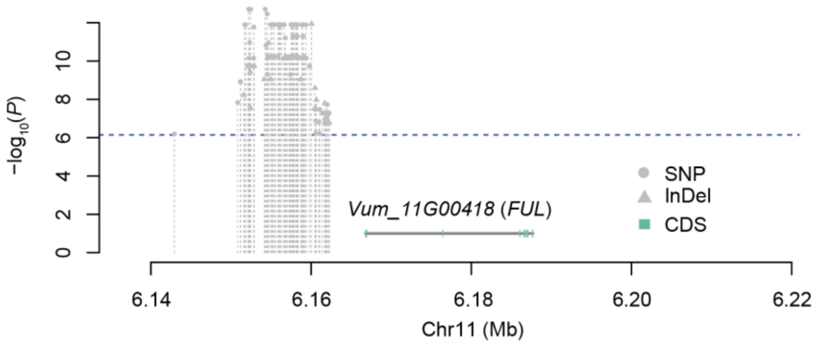

Supplementary Fig. 10. Local Manhattan plot for SNPs and InDels significantly associated with flowering time at the Sanya site within 30 Kb of the candidate gene *FUL*. Red horizontal dashed line indicated the Bonferroni-corrected significance thresholds of GWAS (α = 1).

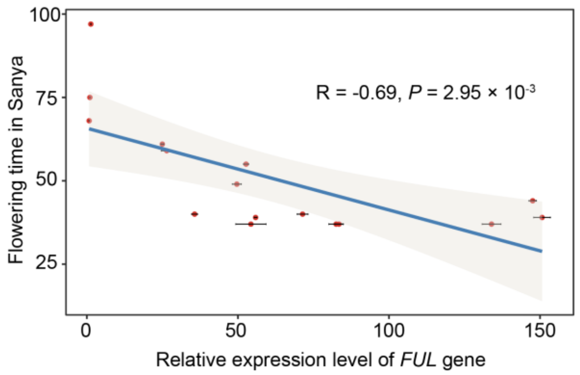

Supplementary Fig. 11. A significantly negative correlation between flowering time and the *FUL* expression level in newly expanded leaves in a panel of 16 diverse rice bean landraces. Data are presented as mean ± SD. Significance was tested with two-sided Student's t-tests.

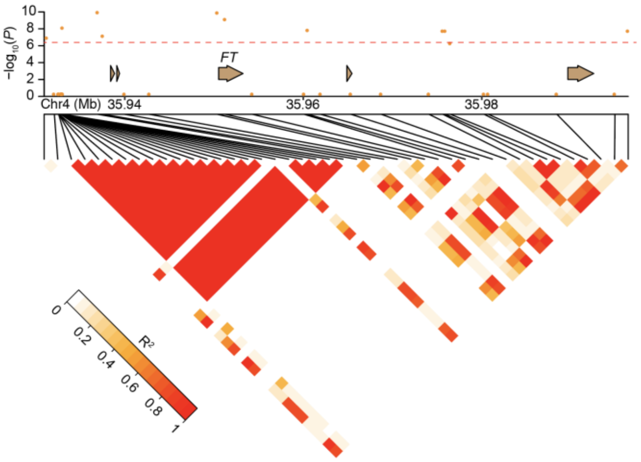

Supplementary Fig. 12. Local Manhattan plot and pairwise linkage disequilibrium (LD) heat map among SNPs located in the associated region with the flowering time measured in Nanning in 2020. The five protein-coding genes are shown as colored arrows. Red horizontal dashed line indicated the Bonferroni-corrected significance thresholds of GWAS (α = 1).

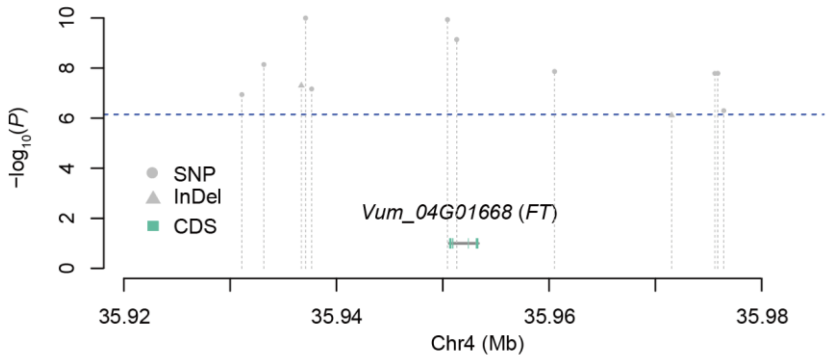

Supplementary Fig. 13. Local Manhattan plot for SNPs and InDels significantly associated with flowering time at the Nanning site within 30 Kb of the candidate gene *FT*. Red horizontal dashed line indicated the Bonferroni-corrected significance thresholds of GWAS (α = 1).

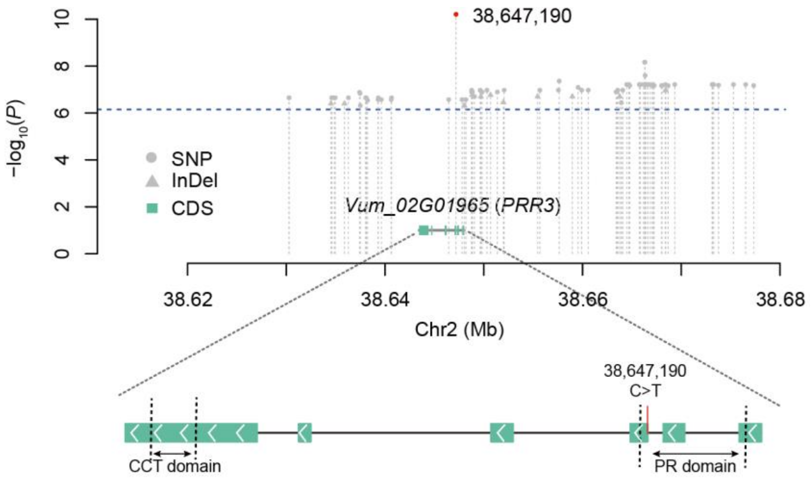

Supplementary Fig. 14. Local Manhattan plot for SNPs and InDels significantly associated with flowering time at the Beijing site within 30 Kb of the candidate gene *PRR3*. The linear gene structure displays the CDS region (rectangles), and introns (horizontal solid grey lines). The white arrow indicates the 5’ to 3’ direction. The red dot and vertical solid lines represent the peak SNP. The dotted lines represented the physical coordinates of the two functional domains (PR, pseudo receiver; CCT, named after the proteins CONSTANS [CO], CO-like, and TOC1, which contain this domain). Red horizontal dashed line indicated the Bonferroni-corrected significance thresholds of GWAS ($\alpha = 1$).

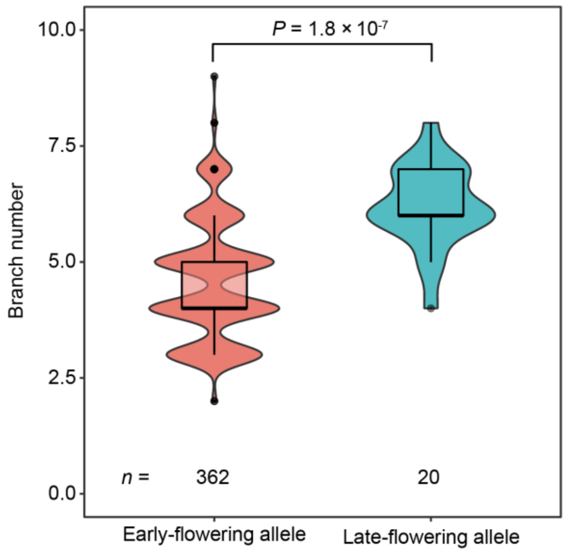

Supplementary Fig. 15. The branch number distribution showed significant differences among landraces carrying the early-flowering *FUL* allele and those carrying the late-flowering *FUL* allele, respectively. The significance was tested with two-sided Wilcoxon tests. In the violin plots, central line: median values; bounds of the box: 25th and 75th percentiles; whiskers: 1.5 * IQR (IQR: the interquartile range between the 25th and 75th percentile). The number (*n*) of landraces carrying each allele is shown below. Source data are provided as a Source Data file.

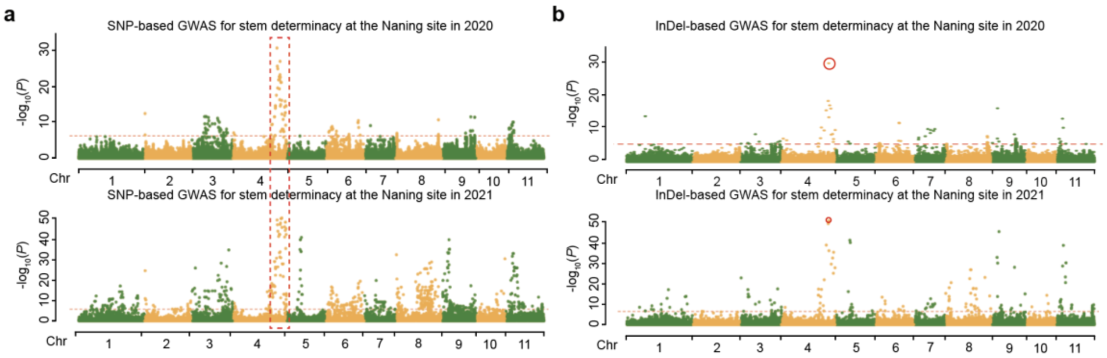

Supplementary Fig. 16. Manhattan plots of SNP-based (a) and InDel-based (b) GWAS for stem determinacy measured at the Nanning site across two years (2020 and 2021). The peak InDel (2-nt deletion) is indicated by the red circle. Red horizontal dashed line indicated the Bonferroni-corrected significance thresholds of GWAS (α = 1).

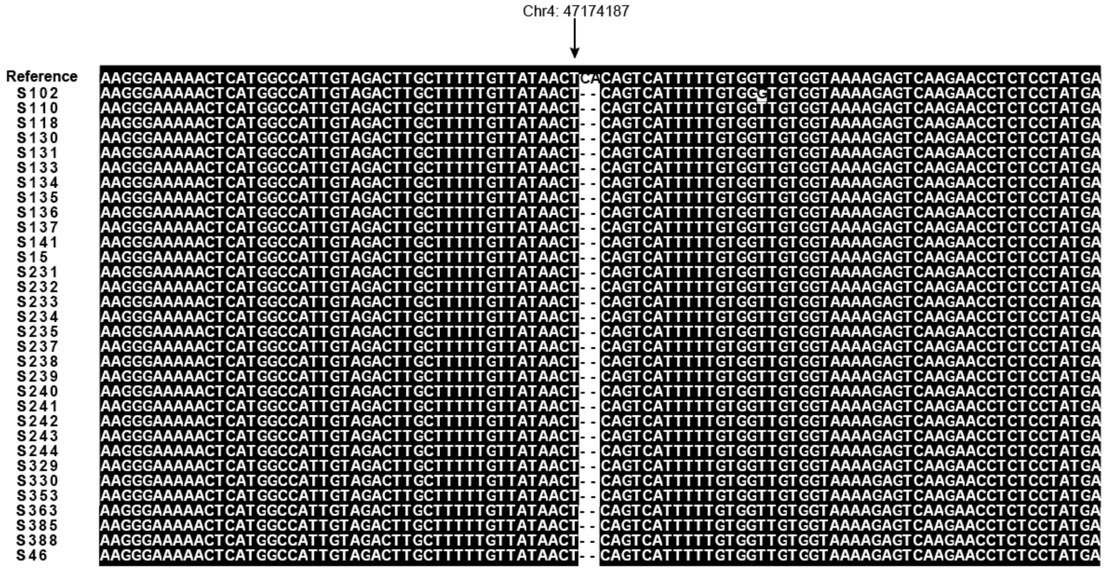

Supplementary Fig. 17. The verification of the homologous 2-bp deletion on the first exon of the *TFL1* gene in the 32 landraces using Sanger sequencing technology.

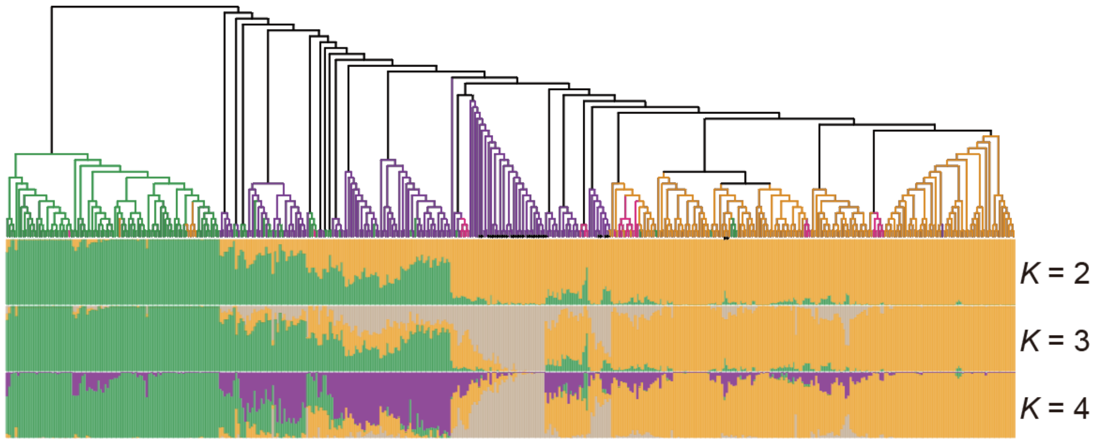

Supplementary Fig. 18. Phylogenetic tree and model-based clustering (*K* = 2–4) of 440 sequenced landraces. The landraces with 2-nt deletion in the *TFL1* gene were indicated by the black triangle on the phylogenetic tree.

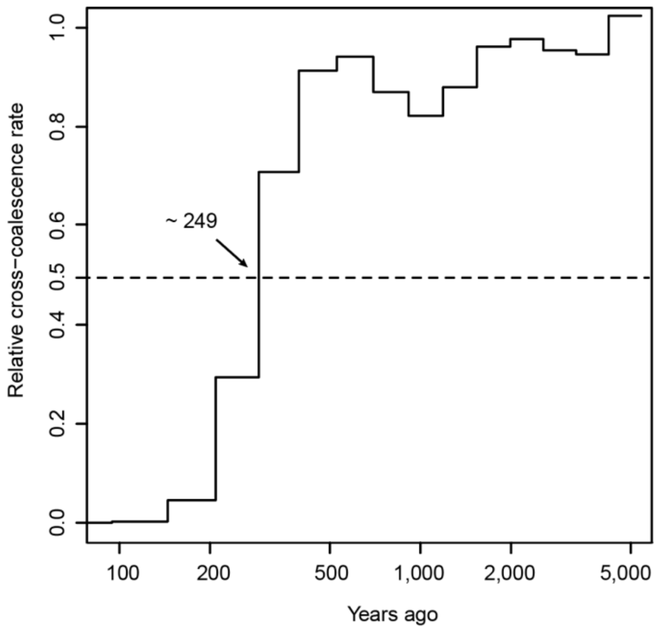

Supplementary Fig. 19. Relative cross coalescence rate between the 32 landraces carrying homozygous mutation (2-bp deletion) alleles and the other landraces in the SC group. The Relative cross coalescence rate was estimated using the MSMC2 program. Values close to 1 indicate that the two populations have not yet diverged. Values close to 0 indicate that the populations have fully diverged. The divergence time was determined under a mutation rate $\mu = 1.5 \times 10^{-8}$ per site per generation when the value was 0.5.

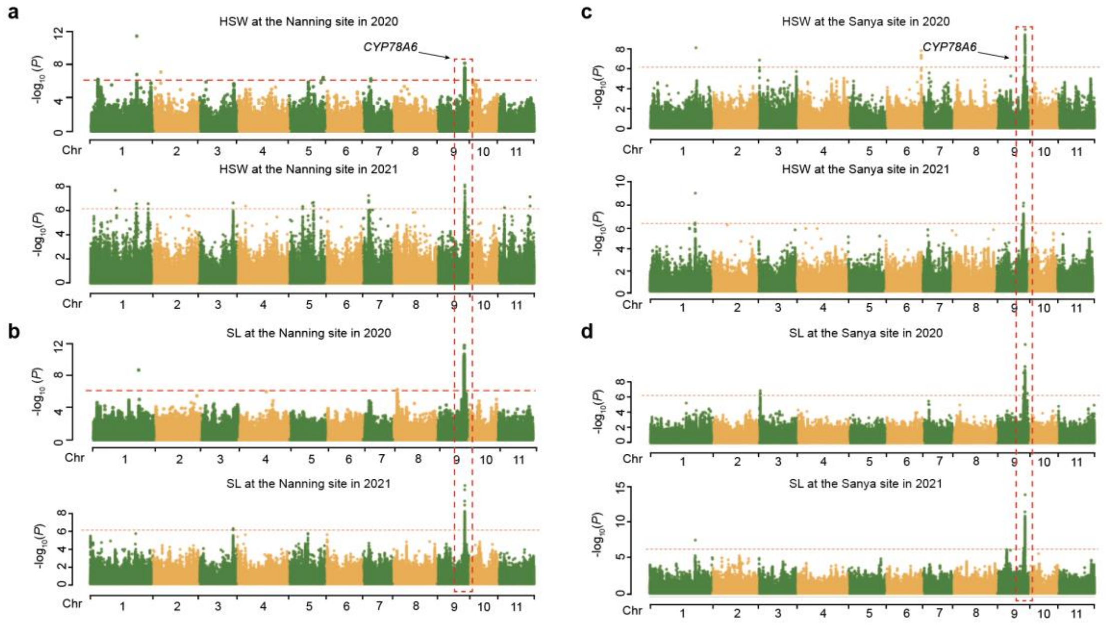

Supplementary Fig. 20. Manhattan plots of GWAS for seed yield traits measured at the Nanning (a-b) and Sanya (c-d) sites across two years (2020 and 2021). HSW, hundred seed weight; SL, seed length. Red horizontal dashed line indicated the Bonferroni-corrected significance thresholds of GWAS ($\alpha = 1$).

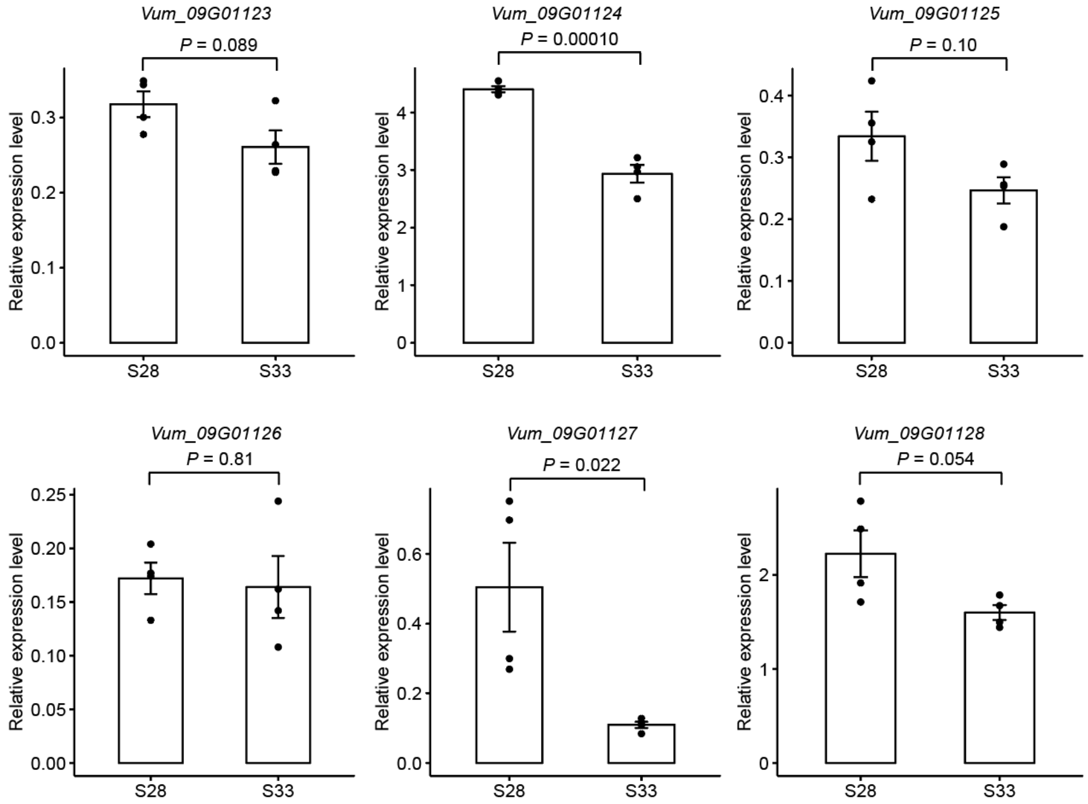

Supplementary Fig. 21. The expression levels of six candidate genes responsible for the association with seed yield component traits. The two genes *Vum_09G01124* and *Vum_09G01127* was located in the associated region (Chr9: 29,030,437 – 29,126,729) and showed differential expression in the seed tissue at 16 DAP between the long-pod (S28) and short-pod landraces (S33), each with four biological replicates. The significance was assessed using two-sided Student's t-test. The data are shown as mean ± SE, and the error bars represent SE. Source data are provided as a Source Data file.

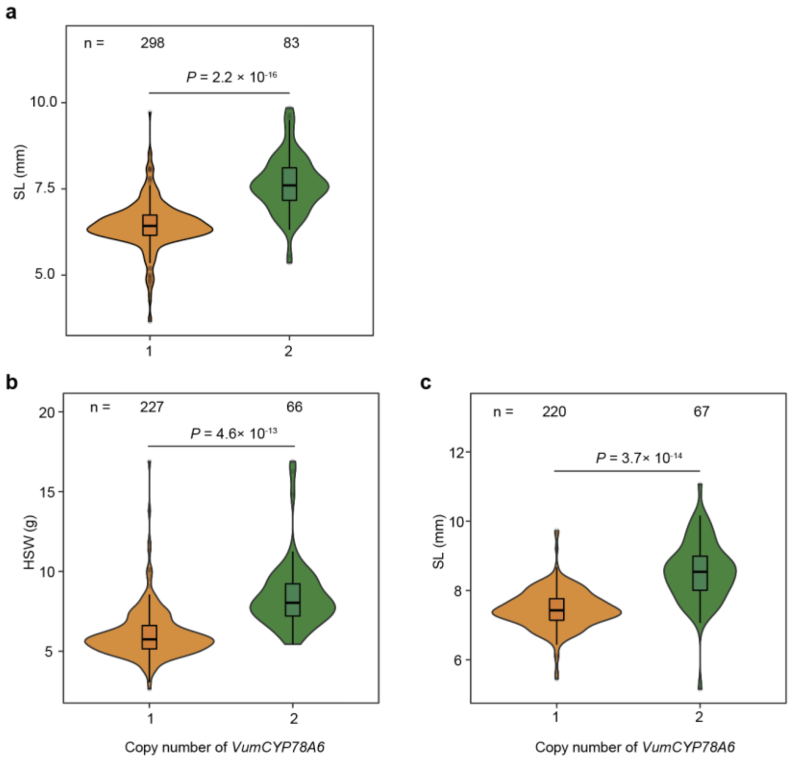

Supplementary Fig. 22. The comparisons of seed yield component traits measured at the Nanning (a) and Sanya sites (b-c) among landraces with different copy numbers of the *VumCYP78A6* gene. HSW, hundred seed weight; SL, seed length. The significance was tested using the two-sided Student's *t*-test. The number (*n*) of landraces with different copy numbers was shown above. In the boxplots, the central line represents the median values, the bounds of the box are corresponding to the 25th and 75th percentiles, and the whiskers represent 1.5 * IQR (the interquartile range between the 25th and 75th percentiles).

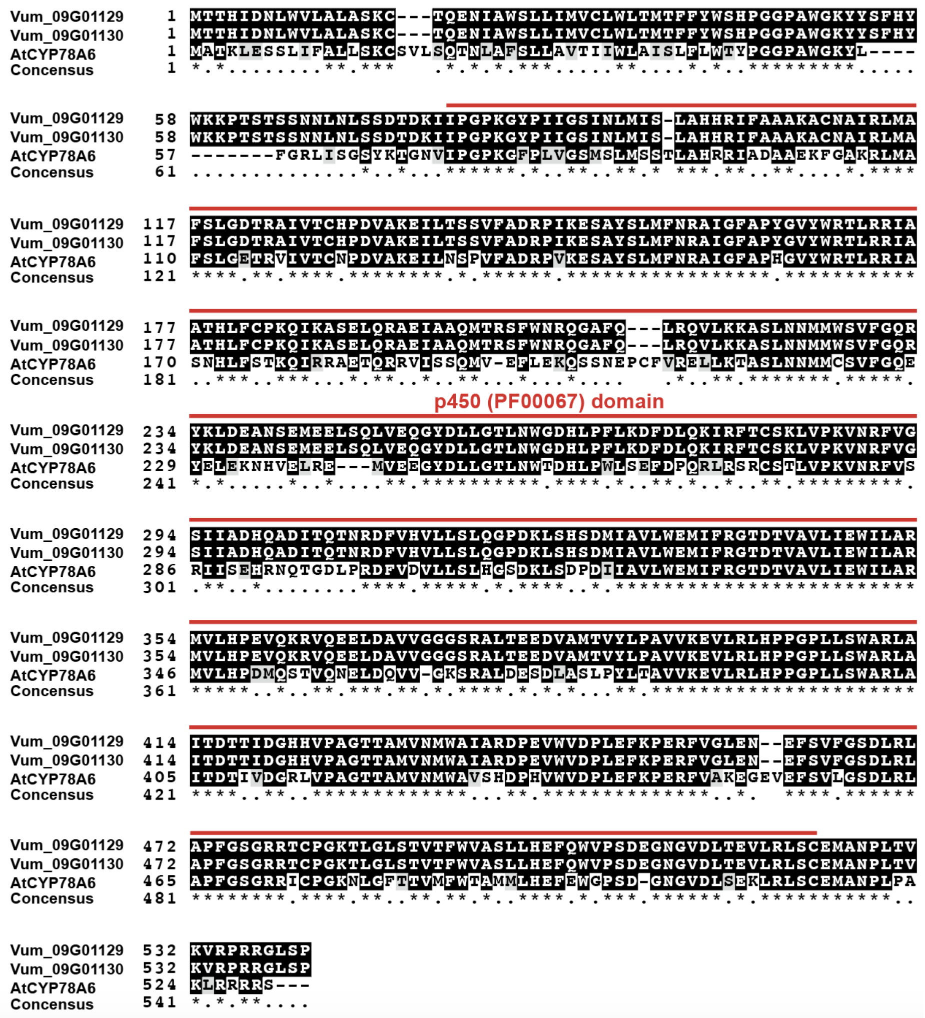

Supplementary Fig. 23. The multiple alignment of protein sequences of *VumCYP78A6-1* (*Vum_09G01129*), *VumCYP78A6-2* (*Vum_09G01129*), and their Arabidopsis ortholog *AtCYP78A6*. The protein sequences below the red lines represented the conserved p450 (PF00067) domain for these three genes.

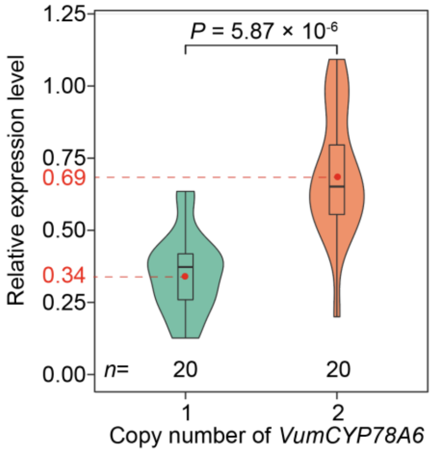

Supplementary Fig. 24. qPCR analysis of seedlings (14 days after sowing) from 20 landraces with one copy of *VumCYP78A6* and 20 landraces with two copies. In the violin plots, central line: median values; bounds of box: 25th and 75th percentiles; whiskers: 1.5 * IQR (IQR: the interquartile range between the 25th and 75th percentile). The red dot represents the average relative expression value. The significance was tested using the two-sided Student's *t*-test. The number (*n*) of landraces carrying different copy numbers is shown below. Source data are provided as a Source Data file.

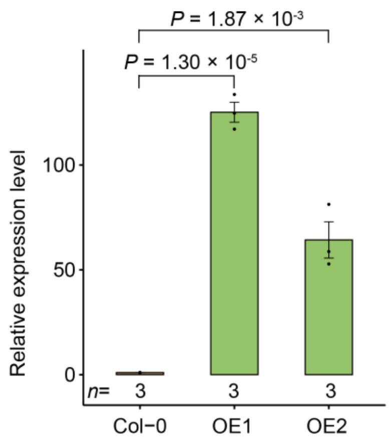

Supplementary Fig. 25. qRT-PCR measurement of the expression levels of *VumCYP78A6-2* in the leaves of wild type (*Arabidopsis thaliana* Col-0) and two independent *VumCYP78A6-2*-OE transgenic plants (OE1 and OE2). Significance was tested using two-sided Student's *t*-test. The data are shown as mean ± SE, and the error bars represent SE. The number (*n*) of each independent experiment is shown below. Source data are provided as a Source Data file.

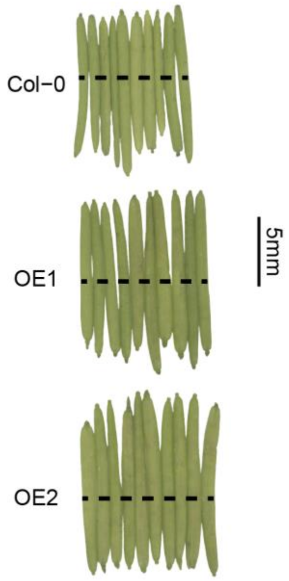

Supplementary Fig. 26. The silique morphology of the wild type (*Arabidopsis thaliana* Col-0) and two independent *VumCYP78A6-2-OE* transgenic lines. Siliques from 6-week-old plants are shown. Bar = 5 mm.

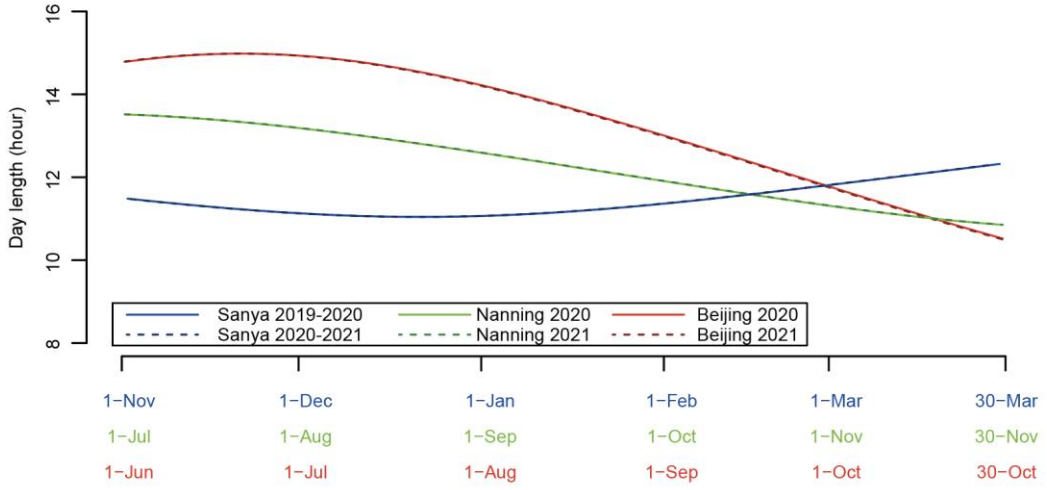

Supplementary Fig. 27. The day length (hour) per day during the growth period at the Sanya site (18° N) in 2019-2020 and 2020-2021, at the Nanning site (22° N) in 2020 and 2021, and at the Beijing site (40° N) in 2020 and 2021.

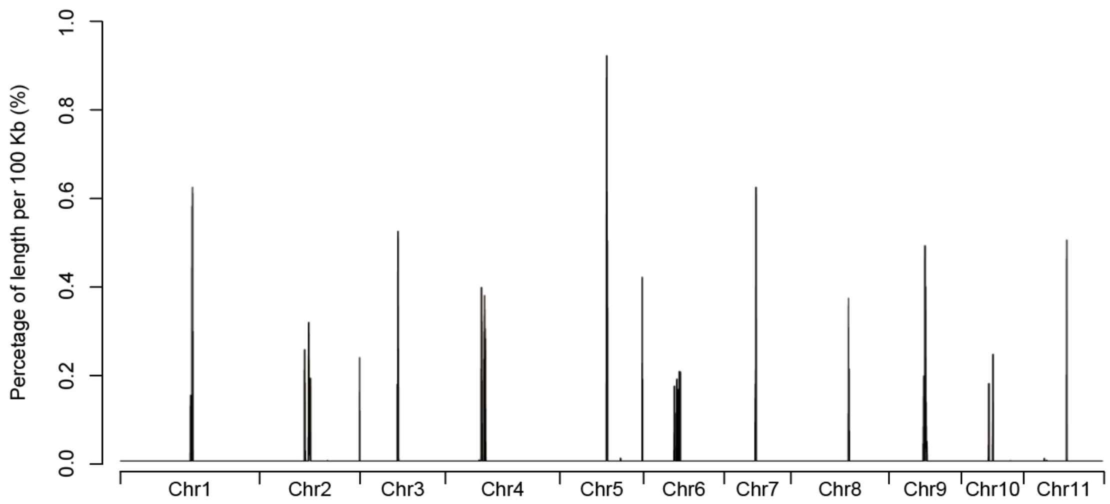

Supplementary Fig. 28. The frequency distribution of the 217-bp centromere-specific repeat.

Supplementary Table 1. Statistics of genome sequencing data for FF25.

**PacBio SMRT**	Read number	10,994,155
	Base number	142,946,051,635
	N50 length (bp)	19475
	Depth	300.54 ×
**Illumina NovaSeq**	Read number	338,192,988
	Base number	50,728,948,200
	Length (bp)	150
	Depth	106.65 ×
**Hi-C**	Read Pair number	465,992,774
	Base number	69,898,916,100
	Length (bp)	150
	Depth	146.96 ×

Supplementary Table 2. Completeness of the FF25 genome assembly.

	Feature	Genome	Protein
BUSCOs	Complete BUSCOs	2,064 (97.3%)	2,057 (96.9%)
	Complete and single-copy BUSCOs	1,965 (92.6%)	1,967 (92.7%)
	Complete and duplicated BUSCOs	99 (4.7%)	90 (4.2%)
	Fragmented BUSCOs	11 (0.5%)	21 (1.0%)
	Missing BUSCOs	46 (2.2%)	43 (2.1%)
	Total	2,121	2,121
LAI		20.3	-

Supplementary Table 3. Distributions of protein-coding genes on chromosomes and unanchored contigs.

Chromosome	Gene number
Chr1	4,221
Chr2	2,684
Chr3	2,122
Chr4	3,300
Chr5	2,164
Chr6	1,984
Chr7	1,882
Chr8	2,769
Chr9	1,613
Chr10	1,927
Chr11	1,764
Contigs	306
Total genes anchored	26,430 (98.86%)
Total	26,736

Supplementary Table 4. Distributions of transcription factor (TF), noncoding RNA genes, and pseudogenes on chromosomes and unanchored contigs.

	TF	microRNA	rRNA	snoRNA	tRNA	Pseudogenes
Chr1	360	146	0	101	110	1,067
Chr2	241	135	4	52	51	901
Chr3	167	100	33	66	51	886
Chr4	259	125	13	77	66	977
Chr5	160	109	6	59	54	781
Chr6	163	92	1	62	47	1,048
Chr7	160	57	0	49	44	534
Chr8	258	128	0	61	57	742
Chr9	122	60	2	38	39	779
Chr10	182	62	414	23	44	581
Chr11	114	104	1	49	26	1,178
Contigs	16	164	84	77	175	161
Genome	2,202	1,282	558	714	764	9,635

Supplementary Table 5. Percentage of the gene with different FPKM ranges in at least one tissue sequenced.

	**Gene number**	**Percentage (%)**
FPKM>1	22,956	85.86
FPKM>10	16,895	63.19
FPKM>100	3,912	14.63

Supplementary Table 6. Number of protein-coding genes annotated by five public database.

	**Number**	**Percentage (%)**
nr	25,990	97.21
InterPro	21,160	79.14
Pfam	20,424	76.39
GO	15,610	58.39
KEGG	2,041	7.63
Total annotated	26,061	97.48

Supplementary Table 7. Summary statistics of syntenic blocks and genes between the Vum genome and its closely related *Vigna* species.

Species in *Vigna* genus	Van	Vst	Vra	Vun
Syntenic block number	639	714	831	803
Number and percentage of syntenic genes in Vum genome	20,985/79.40%	19,459/73.62%	17,980/68.03%	19,990/75.63%

V. angularis, Van; *V. stipulacea*, Vst; *V. radiata*, Vra; *V. unguiculate*, Vun

Supplementary Table 8. Summary of functional annotations for SNPs and InDels.

Functional annotation	Number
**SNPs**	
intergenic	6,406,888
exonic	748,590
intronic	1,935,147
upstream	747,199
downstream	721,248
splicing	1,086
synonymous	740,718
nonsynonymous	351,272
stopgain	4,200
stoploss	404
**InDels**	
intergenic	1,753,129
exonic	36,934
intronic	465,147
upstream	253,788
downstream	244,181
splicing	1,246
frameshift	19,694
nonframeshift	15,530
stopgain	1,038
stoploss	97

Supplementary Table 9. GWAS QTLs detected across two years (2020 and 2021).

Trait	Site	QTL	Years[a]	PVE (%)	Candidate gene
Flowering time	Sanya	Chr8:1,909,300-1,919,793	2	10.57	-
		Chr11:6,142,933-6,162,249	2	14.86	*Vum_11G00418* (*FUL*)
	Nanning	Chr4:35,931,101-35,996,258	2	8.23	*Vum_04G01668* (*FT*)
	Beijing	Chr2:38,647,190-38,647,190	2	17.30	*Vum_02G01965* (*PRR3*)
Stem determinacy	Nanning	Chr3:12,535,769-16,852,788	2	8.69	-
		Chr4:42,022,544-54,020,700	2	41.43	*Vum_04G02513* (*TFL1*)
		Chr5:13,477,451-13,789,216	2	32.19	-
		Chr6:2,759,250-6,161,777	2	12.42	-
		Chr7:18,729,386-20,781,885	2	8.02	-
		Chr9:28,394,569-28,697,545	2	8.24	-
		Chr11:5,078,886-15,461,758	2	24.72	-
HGW	Sanya	Chr9:29,081,975-29,126,729	2	8.88	*Vum_09G01129* &
	Nanning	Chr9:29,030,437-29,126,729	2	15.74	*Vum_09G01130*
Seed length	Sanya	Chr9:29,030,437-29,126,729	2	11.39	(*VumCYP78A6-1* &
	Nanning	Chr9:29,030,437-29,126,729	2	16.17	*VumCYP78A6-2*)

[a] Number of years in which the QTL was detected.

Supplementary Table 10. AIC (akaike information criteria) value of five different genetic models used in the association analysis of the alleles associated with flowering-time data measured in two years at the three distinct sites.

Alleles	Year	AIC				
		Co-dominant	Dominant	Recessive	Over-dominant	Additive
FUL	2020	-147.20	-136.30	-120.10	-35.70	-149.06
	2021	-169.10	-166.50	-146.80	-92.10	-169.82
FT	2020	-8.00	-9.50	32.10	78.00	-2.32
	2021	52.10	50.20	93.40	116.70	61.50
PRR3	2020	-351.00	-347.70	-340.20	-269.10	-352.80
	2021	-356.00	-352.20	-340.50	-272.50	-357.48

Supplementary Table 11. The position and lengths of the potential centromere.

Chr	Length (bp)	Centromere			
		Start (bp)	End (bp)	Length (Mb)	Number of gaps
1	65,796,979	33,160,000	34,360,000	1.20	2
2	47,516,185	21,420,000	24,330,000	2.91	3
3	40,359,536	17,800,000	18,480,000	0.68	1
4	54,125,813	17,210,000	19,520,000	2.31	2
5	39,577,938	22,350,000	23,060,000	0.71	5
6	38,145,303	14,830,000	17,900,000	3.07	2
7	31,513,027	15,040,000	15,710,000	0.67	1
8	46,389,798	27,740,000	28,290,000	0.55	1
9	34,248,148	16,950,000	18,780,000	1.83	1
10	29,464,087	13,510,000	15,910,000	2.40	1
11	38,052,607	21,040,000	21,330,000	0.29	1

Supplementary Table 12. Primers used in this study.

Primer ID	Primer sequences (5'-3')	Purpose
TFL1	Forward: TCTCTCAGATAAGGGTCACT<br>Reverse: CCTCGGTTTCACTCTCTAAA	Verification for the 2-bp InDel in gene *Vum_04G02513* (*TFL1)*
qACT	Forward: CCCCGAAGAACACCCTGTAC<br>Reverse: ACCGCCTGAATTGCCACATA	qRT-PCR for gene Vum_05G00112 (*ACTIN*)
qFUL	Forward: TGTGATGCTCAAGTCGCACT<br>Reverse: CCTATGTGGGCGTGTCTCTC	qRT-PCR for gene *Vum_11G00418* (*FUL*)
qVumCYP78A6	Forward: TTGGCCATAACTGATACCAC<br>Reverse: ACAGAAAACTCGTTCTCCAA	qRT-PCR for tandemly duplicated gene *VumCYP78A6* (*Vum_09G01129* and *Vum_09G01130*)
VumCYP78A6	Forward: ATGACAACGCACATTGATAACCT<br>Reverse: TTAAGGGCTTAGTCCACGCCT	Gene cloning for gene *VumCYP78A6* used in Arabidopsis transformation experiment
