# Supplementary material for: Genomic analyses of rice bean landraces reveal adaptation and yield related loci to accelerate breeding
Source: Nat Commun. 2022 Sep 29;13:5707. doi: 10.1038/s41467-022-33515-2 (PMC9523027; doi:10.1038/s41467-022-33515-2)
Supplement: Supplementary file 3 — Description of Additional Supplementary Files [file 41467_2022_33515_MOESM3_ESM.pdf]

Description of Additional Supplementary Files

File Name: Supplementary Data 1
Description: Protein coding genes in the expanded gene family of rice bean genome.

File Name: Supplementary Data 2
Description: Statistics of repeat sequences in rice bean genome.

File Name: Supplementary Data 3
Description: Summary of re-sequencing data of 440 rice bean landraces.

File Name: Supplementary Data 4
Description: Proteincoding genes within the selective sweep regions in the three comparisons of SSA vs. SC, SSA vs. NC, and SC vs.NC.

File Name: Supplementary Data 5
Description: The information of QTL regions and peak SNP/InDel with its PVE value.

File Name: Supplementary Data 6
Description: The information of candidate genes overlapping with the most significant associated signal of flowering time at the Nanning site.

File Name: Supplementary Data 7
Description: The information of candidate genes overlapping with the most significant associated signal of seed yield component traits.
