# Supplementary material for: Genomic analyses of rice bean landraces reveal adaptation and yield related loci to accelerate breeding
Source: Nat Commun. 2022 Sep 29;13:5707. doi: 10.1038/s41467-022-33515-2 (PMC9523027; doi:10.1038/s41467-022-33515-2)
Supplement: Supplementary file 5 — Reporting Summary [file 41467_2022_33515_MOESM5_ESM.pdf]

Corresponding author(s): Hua Xie, Lixia Wang

Last updated by author(s): 2022-September-18

Reporting Summary

Nature Portfolio wishes to improve the reproducibility of the work that we publish. This form provides structure for consistency and transparency in reporting. For further information on Nature Portfolio policies, see our Editorial Policies and the Editorial Policy Checklist.

Statistics

For all statistical analyses, confirm that the following items are present in the figure legend, table legend, main text, or Methods section.

n/a	Confirmed	
☐	☒	The exact sample size (*n*) for each experimental group/condition, given as a discrete number and unit of measurement
☐	☒	A statement on whether measurements were taken from distinct samples or whether the same sample was measured repeatedly
☐	☒	The statistical test(s) used AND whether they are one- or two-sided<br>*Only common tests should be described solely by name; describe more complex techniques in the Methods section.*
☒	☐	A description of all covariates tested
☐	☒	A description of any assumptions or corrections, such as tests of normality and adjustment for multiple comparisons
☐	☒	A full description of the statistical parameters including central tendency (e.g. means) or other basic estimates (e.g. regression coefficient) AND variation (e.g. standard deviation) or associated estimates of uncertainty (e.g. confidence intervals)
☐	☒	For null hypothesis testing, the test statistic (e.g. *F*, *t*, *r*) with confidence intervals, effect sizes, degrees of freedom and *P* value noted<br>*Give P values as exact values whenever suitable.*
☒	☐	For Bayesian analysis, information on the choice of priors and Markov chain Monte Carlo settings
☒	☐	For hierarchical and complex designs, identification of the appropriate level for tests and full reporting of outcomes
☐	☒	Estimates of effect sizes (e.g. Cohen's *d*, Pearson's *r*), indicating how they were calculated

Our web collection on statistics for biologists contains articles on many of the points above.

Software and code

Policy information about availability of computer code

Data collection	We used Olympus Stream software (v2.4.2) for the measurement of cell numbers in the cross-sections of pod walls. No custom code was used for data collection.
Data analysis	We only used freely software available informatics for our data analysis.<br>Canu (v1.9), GCE (v1.0.2), BUSCO (v3.0.297), Pilon (v1.23), Juicer (v1.8.9), 3D-DNA (v180922), RepeatMasker (v4.1.0), RepeatModeler (v2.0), LTRharvest (v1.5.10), LTR_FINDER (v1.07), AUGUSTUS (v3.2), HISAT (v2.1.0), StringTie (v2.1.0), PASA (v2.5.2), EVidenceModeler (v1.1.1), TransDecoder (v5.5.0), InterProScan (v5.27-66.0), RNAmmer (v1.2), tRNAscan-SEM (v1.23), INFERNAL (v1.1.4), BLAST (v2.5.0+), BWA-MEM (v0.7.17-r1188), Trinity (v2.13.2), BUSCO (v3.0.2), OrthoFinder (v2.3.9), RAxML (v8.2.12), iTAK (v18.12), PAML (v4.9), CAFE (v4.2), WGDi (v0.6.0), NGenomeSyn (v1.37), fastp (v0.20.1), Picard (v1.118), GATK (v4.1.5.0), FastTree2 (v2.1.10), STRUCTURE (v2.3.451), TreeBeST (v1.9.2), smartPCA (v16000), Beagle (v5.1), MSMC2 (v2.1.1), SMC++ (v1.15.2), PopLDdecay (v3.41), VCFtools (v0.1.17), ClusterProfiler (v3.18.0), Fast-LMM (v2.06.20130802), Plink (v1.90b6.10).<br>All the code used for the analyses is freely available. The scripts can be freely and openly accessed at GitHub [https://github.com/guanjiantao-caas/Code-for-rice-bean].

For manuscripts utilizing custom algorithms or software that are central to the research but not yet described in published literature, software must be made available to editors and reviewers. We strongly encourage code deposition in a community repository (e.g. GitHub). See the Nature Portfolio guidelines for submitting code & software for further information.

Data

Policy information about availability of data

All manuscripts must include a data availability statement. This statement should provide the following information, where applicable:

- Accession codes, unique identifiers, or web links for publicly available datasets
- A description of any restrictions on data availability
- For clinical datasets or third party data, please ensure that the statement adheres to our policy

All datasets reported in this study have been deposited in the National Center for Biotechnology Information (NCBI) with the following accession IDs: FF25 genome assembly, JALEER000000000 [https://www.ncbi.nlm.nih.gov/nuccore/JALEER000000000.1]; Raw data for FF25 genome assembly, PRJNA819955 [https://www.ncbi.nlm.nih.gov/bioproject/PRJNA819955]; Raw data for genome sequencing of 440 landraces, PRJNA803965 [https://www.ncbi.nlm.nih.gov/bioproject/PRJNA803965]. The annotation files including predicted CDS and protein sequences generated for FF25 genome assembly have been deposited at Figshare [https://doi.org/10.6084/m9.figshare.19420058]. The online tools and database used in this paper include: Pfam [http://pfam.xfam.org/], InterPro [https://www.ebi.ac.uk/interpro], NR [https://www.ncbi.nlm.nih.gov/refseq/about/nonredundantproteins/], GO [http://geneontology.org], KEGG [https://www.genome.jp/kegg/], FLOweRing Interactive Database [http://www.phytosystems.ulg.ac.be/florid/]. Source data are provided with this paper.

Field-specific reporting

Please select the one below that is the best fit for your research. If you are not sure, read the appropriate sections before making your selection.

☒ Life sciences ☐ Behavioural & social sciences ☐ Ecological, evolutionary & environmental sciences

For a reference copy of the document with all sections, see nature.com/documents/nr-reporting-summary-flat.pdf

Life sciences study design

All studies must disclose on these points even when the disclosure is negative.

Sample size	We collected resequencing data for 440 rice bean landraces from various geographic regions. The sample size was sufficient for population genomic and GWAS analyses based on literature.
Data exclusions	No data were excluded from analysis.
Replication	All experiments in the study have been repeated successfully at least three times.
Randomization	For phenotypic data collection, the measured samples were randomly selected. The accessions were randomly selected for the expression level measurement of the candidate genes.
Blinding	Investigators were blinded at phenotype data collection. For all experimental analysis, investigators were blinded.

Reporting for specific materials, systems and methods

We require information from authors about some types of materials, experimental systems and methods used in many studies. Here, indicate whether each material, system or method listed is relevant to your study. If you are not sure if a list item applies to your research, read the appropriate section before selecting a response.

Materials & experimental systems

n/a	Involved in the study
☒	☐ Antibodies
☒	☐ Eukaryotic cell lines
☒	☐ Palaeontology and archaeology
☒	☐ Animals and other organisms
☒	☐ Human research participants
☒	☐ Clinical data
☒	☐ Dual use research of concern

Methods

n/a	Involved in the study
☒	☐ ChIP-seq
☒	☐ Flow cytometry
☒	☐ MRI-based neuroimaging
